# Evaluating the Psychometric Properties of the 7-Item Persian Game Addiction Scale for Iranian Adolescents

**DOI:** 10.3389/fpsyg.2019.00149

**Published:** 2019-02-05

**Authors:** Chung-Ying Lin, Vida Imani, Anders Broström, Kristofer Årestedt, Amir H. Pakpour, Mark D. Griffiths

**Affiliations:** ^1^Department of Rehabilitation Sciences, The Hong Kong Polytechnic University, Hung Hom, Hong Kong; ^2^Pediatric Health Research Center, Tabriz University of Medical Sciences, Tabriz, Iran; ^3^Department of Nursing, School of Health and Welfare, Jönköping University, Jönköping, Sweden; ^4^Faculty of Health and Life Sciences, Linnaeus University, Kalmar, Sweden; ^5^The Research Section, Region Kalmar County, Kalmar, Sweden; ^6^Social Determinants of Health Research Center, Qazvin University of Medical Sciences, Qazvin, Iran; ^7^International Gaming Research Unit, Psychology Department, Nottingham Trent University, Nottingham, United Kingdom

**Keywords:** adolescent gaming, confirmatory factor analysis, gaming addiction, online addiction, Rasch model

## Abstract

The 7-item Gaming Addiction Scale (GAS) is a brief instrument based on DSM criteria to assess gaming addiction. Although the psychometric properties of the GAS have been tested using classical test theory, its psychometric properties have never been tested using modern test theory (e.g., Rasch analysis). The present study used a large adolescent sample in Iran to test the psychometric properties of the Persian GAS through both classical test and modern test theories. Adolescents (*n* = 4442; mean age = 15.3 years; 50.3% males) were recruited from Qazvin, Iran. In addition to the GAS, all of them completed the following instruments: the nine-item Internet Gaming Disorder Scale–Short Form (IGDS-SF9), Depression Anxiety Stress Scale (DASS), Pittsburgh Sleep Quality Index (PSQI), and a generic quality of life instrument. Two weeks later, all participants completed the GAS again. Confirmatory factor analysis (CFA) and Rasch analysis were used to test the unidimensionality of the GAS. Pearson correlation coefficients were used to test the test-retest reliability, and a regression model was used to test the criterion-related validity of the GAS. Both CFA and Rasch analysis supported the unidimensionality of the GAS. Pearson correlations coefficients showed satisfactory test-retest reliability of the GAS (*r* = 0.78 to 0.86), and the regression model demonstrated the criterion-related validity of the GAS (β = 0.31 with IGDS-SF9; 0.41 with PSQI). Based on the results, the Persian GAS is a reliable and valid instrument for healthcare providers to assess the level of gaming addiction among Persian-speaking adolescents.

## Introduction

Given the rapid evolution of digitized technology worldwide, activities related to internet and smartphone use have become increasingly popular (Yang et al., 2017; Griffiths, 2018; Tsai et al., 2018). Consequently, health and wellbeing problems related to such activities are of concern to healthcare providers (Kuss et al., 2014; Pontes et al., 2015; Lin et al., 2018e; Yang et al., 2018). In relation to growing health concerns, addiction to online and/or offline gaming has received increasing attention (Lemmens et al., 2009; de Palo et al., 2018) because many studies have demonstrated a variety of negative impacts are associated with it, including social anxiety (Weinstein et al., 2015), depression (Sariyska et al., 2015), poor emotional wellbeing (Rücker et al., 2015), and lowered life satisfaction (Macur and Pontes, 2018).

Since the inclusion of Internet Gaming Disorder (IGD) as a tentative disorder in the latest (fifth) edition of the Diagnostic and Statistical Manual of Mental Disorders (DSM-5; American Psychiatric Association, 2013), studies have commonly reported that one of the main IGD symptoms is a persistent preoccupation with online gaming. Therefore, people with IGD feel it difficult to control or cut down the time they spend on games, and may experience negative consequences, including loss of control, deceiving significant others, conflict with family members, social isolation and fatigue, relationship problems, compromising occupational and/or educational activities, disinterest in other activities, using online gaming to escape or relieve a dysphoric mood, withdrawal symptoms, and an increase of daily gaming over time (i.e., tolerance) (King et al., 2013; Ko and Yen, 2014; Petry et al., 2015). Consequently, identifying whether an individual has a gaming addiction is an important topic for healthcare providers.

Internet gaming disorder has been reported among Iranian adolescents. For instance, a recent study on gaming addiction among a relatively large sample of Iranian high school students (*n* = 1020) reported that the prevalence of gaming addiction was 5.3% (Ahmadi et al., 2014). Another study reported that 17% of 564 seventh graders in Iran were classified as being addicted to video games (Zamani et al., 2010) and a smaller study reported that nearly 60% of 210 Iranian adolescents spent at least one or more hours per day playing video games (Mohammadi et al., 2016). Additionally, gaming addiction among Iranian adolescents has been associated with decreased physical and mental health, increased anxiety and sleepiness, and impaired social functioning (Zamani et al., 2009). However, the prevalence estimations of gaming addiction in the aforementioned studies were not based on any standardized instruments using a strong underlying theoretical concept. Therefore, validating a gaming addiction instrument using a theory-driven concept is needed for Iranian adolescents.

One of the developed instruments for assessing gaming addiction (e.g., King et al., 2013; Cho et al., 2014; Pontes and Griffiths, 2017) is the Gaming Addiction Scale (GAS) (Lemmens et al., 2009). Although the GAS has been translated and used among Iranian high school students to assess the relationship between gaming addiction and academic achievement (Haghbin et al., 2013), the Persian GAS used in that study had not gone undergone robust psychometric testing. The GAS was developed based on seven criteria: salience, tolerance, mood modification, withdrawal, relapse, conflict, and problems (Lemmens et al., 2009). The seven criteria are DSM-based and theory-driven (Griffiths, 2005; Griffiths and Davies, 2005). Several studies have illustrated the promising psychometric properties of the GAS (e.g., Gaetan et al., 2014; Baysak et al., 2016; Khazaal et al., 2016; Lemos et al., 2016). More specifically, the unidimensional structure of the GAS has been confirmed using confirmatory factor analyses Confirmatory factor analysis (CFA) (Khazaal et al., 2016); the internal consistency of the GAS was satisfactory (α = 0.85; Khazaal et al., 2016); the concurrent validity of the GAS was supported by its correlation with time spend on games (*r* = 0.549 to 0.576; Lemmens et al., 2009).

It has also been reported that age, gender, and father’s education were correlated with the GAS in a previous study (Pontes, 2017). These variables were used to examine the criterion-related validity of the GAS because previous studies have demonstrated that gaming addiction is associated with poorer mental health (Zamani et al., 2009), poor sleep quality (Lam, 2014), and decreased quality of life (Dolatabadi et al., 2013). More specifically, people who are at risk of gaming addiction are more likely to be irritable and moody, sleep little, have poor sleep hygiene, and withdraw from real life social interactions. Furthermore, when validating the GAS in the present study, another instrument – Internet Gaming Disorder Scale–Short Form (IGDS9-SF) – was included, as well as time spent gaming. This was because the IGDS9-SF assesses a similar concept to the GAS (i.e., problematic gaming) and time spent on game is a commonly used criterion associated with higher GAS scores (e.g., Lemmens et al., 2009). Among all the variables examined, the GAS was anticipated to have stronger associations with IGDS-SF9, sleep quality, and weekly hours spent on gaming online than other criteria because: (i) time spent on gaming, IGDS-SF9, and GAS *assess the concept of gaming*; and (ii) sleep as a health-related behavior has a *direct association with gaming*. More specifically, individuals will sleep less if they spend more time on gaming.

However, to the best of the present authors’ knowledge, all the aforementioned studies only applied psychometric testing using classical test theory rather than modern test theory. Given that the classical test theory is highly sample-dependent, modern test theory provides additional psychometric information using sample-free perspectives (Chang et al., 2014). To date, only one study has applied modern test theory to assess the psychometric properties of the GAS. This study was carried out among Swiss men (Khazaal et al., 2018) and found that all GAS items comprised high discrimination parameters. However, a literature gap exists because Khazaal et al. (2018) only tested the GAS on male adults. Consequently, modern test theory has not been applied to the GAS among females or adolescents.

More specifically, classical test theory has a strong assumption that each observed score (*X*) is a combination of a true score (*T*) and an error term (*E*), where *X* = *T* + *E* (Cappelleri et al., 2014). However, such a strong assumption is hard to achieve in questionnaires using Likert-type or dichotomous scales, and the results derived from classical test theory cannot totally be trusted (Hobart et al., 2007). Consequently, modern test theory (including item response theory and Rasch models) was developed to tackle the limitations of classical test theory. Taking Rasch model as an example, it suggests using a logistic equation (Pi = [exp(𝜃-bi)]/[1 + exp(𝜃-bi)], where Pi denotes the probability that a respondent correctly answers item i, and bi denotes the item difficulty) to calculate the item difficulties for each item and person abilities for each respondent (Wright, 1977). Using such an equation, Rasch model uses the probabilities in responding to an item to convert a dichotomous scale score into a continuous score with a standard unit of *logit* (Chang et al., 2015). Therefore, in addition to psychometric testing using classical test theory, the present study applied one type of the modern test theory (i.e., Rasch analysis) to evaluate the psychometric properties of the GAS. More specifically, the present authors agree with Lin et al. (2018b) that “the nature of scientific inquiry is to accumulate evidence using different methods” (p.2). Consequently, the GAS should be examined using different statistical methods (i.e., Rasch analysis in the present study) across different populations. More specifically, the psychometric results derived from classical test theory in the present study were performed to replicate and compare to previous findings on the psychometric properties of the GAS (e.g., Lemmens et al., 2009). Psychometric findings derived from modern test theory in the present study were conducted to extend our knowledge concerning the instrument’s psychometric properties (i.e., psychometric information of the GAS in a sample-free method). In brief, the following properties of the GAS were examined using the classical test theory: ceiling and floor effects, internal consistency, corrected item-total correlation, test-retest reliability, CFA, measurement invariance using multi-group CFA (MGCFA), latent class analysis (LCA), and criterion-related validity. The following properties of the GAS were examined using the Rasch model: item statistics, item and person separation reliability, item and person separation index, and differential item functioning (DIF).

## Materials and Methods

The study was approved by the Institutional of Review Board in Qazvin University of Medical Sciences (Iran). All the participants were clearly informed regarding the study purpose and their right to withdraw during the study period. All the participants who were willing to participate signed a written informed consent, as did the parents of the participants.

### Design, Participants, and Recruitment Procedure

In the present study, data were collected from high schools in Qazvin (Iran). The participants included 4442 adolescents from 45 high schools using two-stage stratified cluster random sampling. Initially a list of high schools was obtained from the Organization for Education at Qazvin. Following this, 45 schools were selected randomly to participate in the present study. Of these, 43 schools agreed to participate. From each school, four classes were selected randomly and all adolescents were invited to participate in this study after giving information about the study’s objectives and checking inclusion criteria. The inclusion criteria were simply being aged from 13 to 18 years old and agreeing to participate in the study. Initially, 4794 adolescents were approached, and 352 declined to participate or did not meet the inclusion criteria. The remaining 4442 participants completed the measures in a classroom setting with each participant assigned a special code to identify them for the re-test two weeks later (with a total of 4131 participants completing the retest). Two open-ended questions were used to assess the weekly time spent on gaming: *“How many hours do you spend playing video games on specific platforms (i.e., PCs, consoles, handheld gaming devices) on a school day?”* and *“How many hours do you spend playing video games on specific platforms (i.e., PCs, consoles, handheld gaming devices) on a weekend day?”* To calculate total weekly hours spent on playing video game, the adolescents’ responses on weekday were multiplied by five and the responses on weekend day were multiplied by two. Table 1 shows the characteristics of the participants. More specifically, the mean age of the participants (*N* = 4442) was 15.3 years (*SD* = 1.6) with slightly more than half being male (*n* = 2236; 50.3%). Less than one-third of the participants (*n* = 1403; 31.6%) were current smokers. On average, the participants spent 18.9 h (*SD* = 5.6) gaming online per week.

**Table 1:** Participants characteristics (*N* = 4442).

	Mean ± SD or *n* (%)
Age (Year)	15.3 ± 1.6
Gender (Male)	2236 (50.3)
Fathers’ education^a^	7.9 ± 4.1
Mothers’ education^a^	6.6 ± 3.8
Current smoker (Yes)^b^	1403 (31.6)
7-item Gaming Addiction Scale score	2.6 ± 0.7
Internet Gaming Disorder Scale-Short Form	24.6 ± 8.3
Depression^c^	7.3 ± 4.1
Anxiety^c^	7.9 ± 4.6
Stress^c^	7.9 ± 4.5
Pittsburgh Sleep Quality Index	4.7 ± 2.8
PedsQL^TM^ 4.0 SF15	76.8 ± 14.5
Weekly hours spent gaming online (hours)	18.9 ± 5.6

*^*a*^Number of years spent in full-time education by fathers or mothers. ^*b*^38 participants did not report their smoking status. ^*c*^Measured using Depression Anxiety Stress Scales. PedsQL^*TM*^ 4.0 SF15 = Pediatric Quality of Life Inventory Short Form.*

### Translation Procedure

The translation of the GAS was conducted in several steps, based on recommendations from international guidelines (Beaton et al., 2000; Wild et al., 2005; Pakpour et al., 2011). Forward translation was performed by two bilingual translators whose mother tongue was Persian/Farsi. The two translated version were compared and synthesized into a mutually agreed version in a session with translators as well as a project manager. The provisional forward translation was then back translated into English by two independent translators who were unaware of the original English version of the GAS. All translated versions were reviewed by an expert panel (including a psychiatrist, nurses, a psychologist, a pediatrician, and a psychometrician) to achieve equivalence between both English and Persian versions. The interim Persian version of the GAS was then piloted on 37 adolescents (13 boys and 24 girls; mean age 16.2 years). The adolescents were also interviewed to explore their thoughts about each questionnaire item and their responses to ensure that both English and Persian versions were equivalent. Through the pilot testing, some words were changed to increase readability for adolescents (e.g., *“Did you spend increasing amounts of time on games?”* was changed to *“Did you spend more and more time on games?”*). The final Persian version of the GAS was then administered to 4442 adolescents to evaluate its psychometric properties.

### Instruments

#### Game Addiction Scale (GAS)

The GAS has a long version with 21 items and a short version with seven items. In the present study, the 7-item GAS was used because its brevity can ease the burden of survey fatigue among respondents. The seven items in the GAS are rated using a five-point Likert scale ranging from 1 (*never*) to 5 (*very often*). A higher score on the GAS indicates more problematic use of online gaming (Lemmens et al., 2009). Moreover, the GAS has been validated using classical test theory in French, Brazilian, German, and Turkish versions (Gaetan et al., 2014; Baysak et al., 2016; Khazaal et al., 2016; Lemos et al., 2016). More specifically, the unidimensional structure of the GAS has been confirmed using CFA (Khazaal et al., 2016). Here, the internal consistency of the GAS was satisfactory (α = 0.85; Khazaal et al., 2016), and the concurrent validity of the GAS was supported in another study by its correlation with time spent on games (*r* = 0.549 to 0.576; Lemmens et al., 2009). Moreover, previous studies using the GAS have classified gamers into four types: addicted gamers (scoring three or more on four core items [relapse, withdrawal, conflict and problems]), problem gamers (scoring three or more on two or three of the four same items), engaged gamers (scoring three or more on the first three items [salience, tolerance, and mood modification] but who did not score three or above on the other items), and normal gamers (gamers not classified as addicted, problem, or engaged) (Brunborg et al., 2015; Wittek et al., 2016).

#### Internet Gaming Disorder Scale–Short Form (IGDS-SF9)

The IGDS-SF9 comprises nine items, which are in accordance with the IGD criteria described in the DSM-5 (Pontes and Griffiths, 2015). A five-point Likert scale ranging from 1 (*never*) to 5 (*very often*) is used for all the items, and a higher score indicates a higher likelihood of IGD. The psychometric properties of the Persian IGDS-SF9 are satisfactory (Wu et al., 2017). More specifically, the internal consistency for the IGDS-SF9 was 0.90 (Wu et al., 2017), and the concurrent validity of the IGDS-SF9 was supported by the strong correlation with time spent playing on online games (*r* = 0.62; Wu et al., 2017).

#### Depression Anxiety Stress Scale (DASS)

The Depression Anxiety Stress Scale (DASS) comprises 21 items comprising three subscales (i.e., depression, anxiety, and stress). A four-point Likert scale ranging from 0 (*did not apply to me at all*) to 3 (*completely applied to me*) is used for all the items, and a higher score indicates a higher level of depression, anxiety, or stress (Lovibond and Lovibond, 1995). The psychometric properties of the Persian DASS are satisfactory (Asghari et al., 2008; Lin et al., 2017). More specifically, the internal consistency for the DASS is very good to excellent (between 0.84 and 0.91; Lin et al., 2017), and the concurrent validity of the DASS has been supported by the strong correlation with the four Systems Anxiety Questionnaire and Beck Depression Inventory (*r* = 0.40 to 0.70; Asghari et al., 2008).

#### Pittsburgh Sleep Quality Index (PSQI)

The Pittsburgh Sleep Quality Index (PSQI) comprises 19 items to assess sleep quality and disturbance. A four-point Likert scale ranging from 1 (*not during the past month*) to 4 (*three times a week or more*) is used for all the items, and a higher score indicates poorer sleep quality. The psychometric properties of the Persian PSQI are satisfactory (Moghaddam et al., 2012). More specifically, the internal consistency for the PSQI is very good to excellent (0.89 and 0.91; Lin et al., 2018e), and the concurrent validity of the PSQI has been supported by the strong correlation with General Health Questionnaire-12 (*r =* 0.54; Moghaddam et al., 2012).

#### Pediatric Quality of Life Inventory Short Form (PedsQL^TM^ 4.0 SF15)

The PedsQL^TM^ 4.0 SF15 comprises 15 items distributed across four subscales (i.e., physical, emotional, social, and school). A five-point Likert scale ranging from 0 (*never a problem*) to 4 (*almost always a problem*) is used for all the items, and a higher score indicates a better quality of life (Varni et al., 2001). The psychometric properties of the Persian PedsQL^TM^ 4.0 SF15 are satisfactory (Pakpour, 2013). More specifically, the internal consistency for the PedsQL 4.0 SF15 is good (0.82; Pakpour, 2013), and the known-group validity of the PedsQL^TM^ 4.0 SF15 has been supported by the significantly different scores found between schoolchildren and pediatric patients (*p <* 0.05; Pakpour, 2013).

### Statistical Analysis

The statistics were analyzed using SPSS (for descriptive statistics), MPLUS (for CFA and LCA), and WINSTEP (for Rasch analysis).

#### Psychometric Evaluation Under Classical Test Theory

The ceiling and floor effects of the GAS were first examined, and a negligible effect was proposed at 3%, a minimal effect at 5%, a moderate effect at 15%, and a substantial effect at 60% (Lin et al., 2013). The Cronbach’s α was then computed where >0.7 indicates satisfactory internal consistency (Nunnally and Bernstein, 1994; Lin et al., 2018a). Alongside the Cronbach’s α, the corrected item-total correlation was calculated and where a value of >0.4 is deemed as acceptable (Wang et al., 2007). Standard error of measurement was further calculated using the Cronbach’s α and SD of the GAS score, and a low value is in anticipation (Su et al., 2014). Following this, a Pearson correlation was conducted to examine the test-retest reliability of the GAS where a value >0.75 represents excellent (Lin et al., 2012).

Confirmatory factor analysis using diagonally weighted least squares (WLSMV) estimation was used to examine the hypothesized one-factor structure of the GAS (i.e., unidimensionality), and the factor loadings and uniqueness values derived from the CFA were additionally used to calculate average variance extracted (acceptable value >0.5) and composite reliability (acceptable value >0.6; Fornell and Larcker, 1981; Bagozzi and Yi, 1988). Additionally, comparative fit index (CFI), Tucker-Lewis index (TLI), root mean square error of approximation (RMSEA), and standardized root mean square residual (SRMR) were applied to determine whether the GAS fitted a unidimensional model. The recommended cutoff points were CFI and TLI > 0.9; SRMR and RMSEA < 0.08 (Hu and Bentler, 1998; Cheng et al., 2016). After confirming the unidimensional structure of the GAS, MGCFA was conducted using three nested models to examine the measurement invariance of the GAS across gender groups and groups with different time spent gaming online. The three nested models were a configural model (Model 1), a metric invariance model (Model 2), and a scalar invariance model (Model 3). Model 1 freely estimated the item loadings and item thresholds for each group without any constraints; Model 2 based on Model 1 to constrain the item loadings being equal across groups; Model 3 based on Model 2 to constrain the item thresholds being equal across groups (Millsap and Yun-Tien, 2004; Bagheri et al., 2014). The comparisons between the groups generated the following fit indices designed to understand whether the measurement invariance was supported: a non-significant χ^2^ test, ΔCFI < |0.01|, ΔSRMR < |0.01|, and ΔRMSEA < |0.015| supports measurement invariance (Chen, 2007).

Criterion-related validity was assessed using structural equation modeling (SEM) with external criteria (including IGDS-SF9, DASS, PSQI, PedsQL^TM^ 4.0 SF15 scores, and weekly hours spent gaming online) to demonstrate the associations between the GAS and these external criteria. More specifically, the GAS was treated as the dependent variable; IGDS-SF9, depression, anxiety, stress, PSQI, PedsQL^TM^ 4.0 SF15, and weekly hours spent gaming online were treated as independent variables; age, gender, and father’s education were treated as controlled variables in the SEM model.

Latent class analysis was used to classify the participants into different groups based on a person-centered approach (Wartberg et al., 2015). The following statistics were used to determine the most appropriate number of groups among the participants: the Akaike information criterion (AIC), the Bayesian information criterion (BIC), the sample-size adjusted Bayesian information criterion (SSABIC), Lo-Mendell-Rubin’s likelihood ratio test (LMR), and entropy with Bootstrap Likelihood Ratio Test. A lower value in AIC, BIC, and SSABIC suggests better-fitting models; a higher value in the entropy demonstrates a better classification quality. In addition, LMR compares the improvement between models (i.e., a significant *p*-value from the LMR indicates a model outperforms its previous model). After determining the group number, a multinomial logistic regression was used to investigate the association between each predictor/covariate and class membership. More specifically, the lowest class was treated as the reference group to see whether participants classified as having higher addiction risk had higher odds ratio (OR) in age, male, father’s education, IGDS-SF9, depression, anxiety, stress, and PSQI.

#### Psychometric Evaluation Under Rasch Analysis

Rasch analysis was conducted using a partial credit model, and the unit of *logit* was generated to report item difficulty for each item. In addition, information-weighted fit statistic (infit) mean square (MnSq) and outlier-sensitive fit statistic (outfit) MnSq were generated in the Rasch analysis to indicate the item properties. More specifically, a good fit of an item should have both infit and outfit MnSq between 0.5 and 1.5, where a value lower than 0.5 indicates too much redundancy and a value higher than 1.5 indicates too much misfit (Jafari et al., 2012; Khan et al., 2013). Rasch analysis also generated item and person separation reliability (acceptable value >0.7; Chang et al., 2014); item and person separation index (acceptable value >2; Chang et al., 2014). Local independence was tested using the correlation matrix between item residuals, and an *r* < 0.3 suggests that local independence is supported.

Item information function (IIF) and test information function (TIF) were illustrated to show how much information can be obtained in the GAS items among which levels of respondents’ ability. More specifically, the IIF illustrates the information for each GAS item; TIF illustrates the information for entire GAS. Evaluation of DIF was conducted using the item difficulties derived from the Rasch models to examine measurement invariance across groups at item level. That is, DIF tests the measurement invariance for each GAS item. Therefore, DIF specifically identifies which items for one group compared to another (e.g., males vs. females) are easier or harder for them to respond to (Lin et al., 2018c,d). Moreover, the difference of item difficulties between groups is presented using DIF contrast, and a commonly used cutoff is >0.5 indicating substantial DIF (Shih and Wang, 2009).

## Results

### Psychometric Findings From Classical Test Theory

#### Item Properties

The means of item scores on the GAS were between 2.48 and 2.78. The items also demonstrated promising properties in terms of their factor loadings (ranging between 0.66 and 0.86), corrected item-total correlations (ranging between 0.72 and 0.84), and test-retest reliability coefficients (ranging between 0.78 and 0.86; Table 2).

**Table 2:** Psychometric properties of the Game Addiction Scale at item level.

Item #	Item score	Analyses from classical test theory			Analyses from Rasch				
	Mean (SD)	Factor loading^a^	Item-total correlation	Test-retest reliability^b^	Infit MnSq	Outfit MnSq	Difficulty	DIF contrast across gender^cd^	DIF contrast across time on gaming^ce^
GAS-1	2.57	0.76	0.76	0.78	1.02	0.98	0.40	–0.23	–0.40
	(0.92)	
GAS-2	2.67	0.86	0.84	0.79	0.76	0.70	0.17	0.20	0.01
	(0.72)	
GAS-3	2.48	0.79	0.79	0.80	0.91	0.86	0.59	0.01	–0.28
	(0.69)	
GAS-4	2.61	0.80	0.82	0.85	0.81	0.83	0.30	–0.03	0.17
	(0.73)	
GAS-5	2.54	0.69	0.72	0.78	1.10	1.14	0.44	–0.14	–0.48
	(0.60)	
GAS-6	2.78	0.77	0.80	0.86	0.92	0.87	–0.08	0.12	0.06
	(0.74)	
GAS-7	2.53	0.66	0.73	0.81	1.04	1.01	–0.82	0.15	0.29
	(0.65)	

*^*a*^Based on confirmatory factor analysis. ^*b*^Using Pearson correlation. ^*c*^DIF contrast >0.5 indicates substantial DIF. ^*d*^DIF contrast across gender = Difficulty for females-Difficulty for males. ^*e*^DIF contrast across time on gaming = Difficulty for participants with median weekly hours or below on gaming (i.e., ≤19 h)-Difficulty for participants with above median weekly hours on gaming (i.e., ≥20 h). MnSq, mean square error; DIF, differential item functioning.*

#### Scale Properties in General

The entire GAS had excellent psychometric properties as indicated by the low ceiling (3.8%) and floor effects (0.9%), high internal consistency (Cronbach’s α = 0.89), satisfactory fit indices in the CFA (CFI = 0.962, TLI = 0.942, RMSEA = 0.078, and SRMR = 0.046), acceptable average variance extracted and composite reliability (coefficients = 0.58 and 0.91, respectively), small standard error of measurement (2.41), and excellent test-retest reliability (*r* = 0.83; Table 3).

**Table 3:** Measurement invariance across gender and across weekly hours spent on gaming online examined using confirmatory factor analysis.

Model and comparisons	Fit indices							
	χ^2^ (df)	Δχ^2^ (Δdf)	CFI	ΔCFI	SRMR	ΔSRMR	RMSEA	ΔRMSEA
**Gender**								
M1: Configural	577.57 (28)^∗^		0.944		0.040		0.066	
M2: Plus all loadings constrained	589.40 (35)^∗^		0.939		0.038		0.065	
M3: Plus all thresholds constrained	601.32 (42)^∗^		0.946		0.036		0.063	
M2–M1		11.83 (7)		–0.005		–0.002		–0.001
M3–M2		11.92 (7)		0.007		–0.002		–0.002
**Weekly hours spent gaming online^a^**								
M1: Configural	460.66 (28)^∗^		0.955		0.030		0.063	
M2: Plus all loadings constrained	468.19 (35)^∗^		0.959		0.029		0.061	
M3: Plus all thresholds constrained	480.22 (42)^∗^		0.961		0.027		0.058	
M2–M1		7.53 (7)		0.004		–0.001		–0.002
M3–M2		12.03 (7)		–0.002		–0.002		–0.003

*^∗^*p* < 0.05. ^*a*^Median weekly hours were 20 h. M1 = Model 1, a configural model; M2 = Model 2, a model based on M1 with all factor loadings constrained being equal across groups; M3 = Mode 3, a model based on M2 with all item thresholds constrained being equal across groups. CFI, comparative fit index; SRMR, standardized root mean square residual; RMSEA, root mean square error of approximation.*

#### Scale Properties in Measurement Invariance

The MGCFA indicated that the measurement invariance of the GAS was supported by all the fit indices, including the non-significant χ^2^ test, in the nested models (Table 3).

#### Scale Properties in Criteria-Related Validity

All external variables assessed (including PedsQL^TM^ 4.0 SF15, IGDS-SF9, depression, anxiety, stress, PSQI, and weekly hours spent gaming online) for testing the criterion-related validity of the GAS were significantly associated with the GAS. In addition, the strongest associations were found with scores on the IGDS-SF9 (standardized coefficient = 0.403), followed by PSQI scores (standardized coefficient = 0.206), and weekly hours spent gaming online (standardized coefficient = 0.112; Table 4).

**Table 4:** Criterion-related validity of the Game Addiction Scale using structural equation modeling (SEM).

Criterion	B (SE)	β	*p*-value
Age^a^	0.05 (0.007)	0.049	<0.001
Gender (male)^a^	0.176 (0.018)	0.120	<0.001
Father’s education^a^	0.013 (0.002)	0.076	<0.001
IGDS-SF9	1.04 (0.001)	0.403	<0.001
Depression^b^	0.006 (0.003)	0.04	0.045
Anxiety^b^	0.024 (0.003)	0.157	<0.001
Stress^b^	0.011 (0.002)	0.052	<0.001
PSQI	0.753 (0.001)	0.206	<0.001
PedsQL^TM^ 4.0 SF15	–0.147 (0.003)	–0.100	<0.001
Weekly hours spent gaming online	0.299 (0.002)	0.112	<0.001

*^*a*^Age, gender, and father’s education (number of years spent in full-time education) were controlled variables in the SEM model. ^*b*^Depression, anxiety, and stress were measured using Depression Anxiety Stress Scales. IGDS-SF9, Internet Gaming Disorder Scale-Short Form; PSQI, Pittsburgh Sleep Quality Index; PedsQL^*TM*^ 4.0 SF15, Pediatric Quality of Life Inventory Short Form. Fit indices: χ^2^ (df) = 1937.554 (110); *p* < 0.001, comparative fit index = 0.956, Tucker-Lewis index = 0.945, root mean square error of approximation: 0.061, standardized root mean square residual: 0.066.*

#### Scale Properties in LCA

**FIGURE 1:**
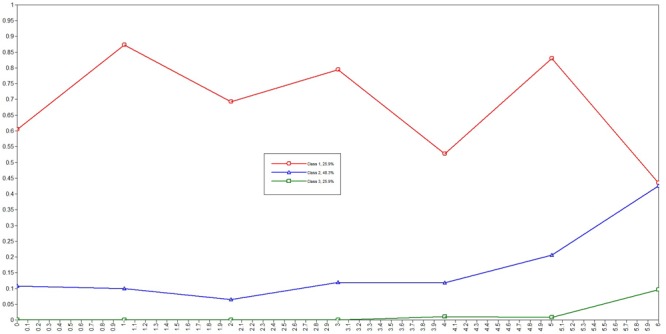
Estimated probabilities for each of the three classes (i.e., class 1: high-addiction risk group; class 2: medium-addiction risk group; and class 3: low-addiction risk group).

The LCA helped classify the participants into three different levels of gaming addiction (Table 5), and they were grouped into a low-addiction risk, a medium-addiction risk, or a high-addiction risk group. The estimated probabilities for each of the three classes are presented in Figure 1. Additionally, it was found that compared to the low-addiction risk group, the medium-addiction risk group were more likely to be older (OR = 1.25; 95% CI = 1.16, 1.34), be a male (OR = 2.49; 95% CI = 2.03, 3.06), have a higher score on the IGDS-SF9 (OR = 3.36; 95% CI = 2.97, 3.79), have higher level of anxiety (OR = 1.04; 95% CI = 1.03, 1.06) and stress (OR = 1.02; 95% CI = 1.00, 1.04), and have poorer sleep quality (OR = 2.62; 95% CI = 2.39, 2.87). Similar findings were found in comparison between high-addiction risk and low-addiction risk groups: OR (95% CI) = 1.42 (1.31, 1.55) for age; 7.06 (5.66, 8.82) for gender; 6.69 (5.86, 7.64) for IGDS-SF9 score; 1.11 (1.09, 1.13) for anxiety; 1.05 (1.03, 1.07) for stress; and 5.56 (4.94, 6.26) for sleep quality (Table 6).

**Table 5:** Latent class analysis to identify subgroups of adolescents.

Model	AIC	BIC	SSABIC	Entropy	L-M-R test (*P*-value)
One-class	86429.266	86589.238	86509.798	–	–
Two-class	75790.130	76116.472	75954.414	0.859	10642.401 (<0.001)
**Three-class**	**71474.264**	**71966.976**	**71722.300**	**0.888**	**4347.001 (<0.001)**
Four-class	70245.023	70904.105	70576.812	0.863	1275.400 (0.763)

*AIC, Akaika’s information criterion; BIC, Bayesian information criterion; SSABIC, sample-size adjusted BIC; L-M-R test, Lo-Mendell-Rubin’s likelihood ratio test. The bold values indicate the best solution in identifying the number of group by latent class analysis.*

**Table 6:** Comparisons among subgroups of adolescents in different risk of gaming addiction.

	Low-addiction	Medium-addiction	High-addiction
	risk	risk	risk
	(*n* = 1159)	(*n* = 2128)	(*n* = 1155)
		OR (95% CI)	OR (95% CI)
Age in year	REF	1.25 (1.16–1.34)^∗∗^	1.42 (1.31–1.55)^∗∗^
Gender (Ref: female)	REF	2.49 (2.03–3.06)^∗∗^	7.06 (5.66–8.82)^∗∗^
Father’s education	REF	0.99 (0.98–1.02)	1.14 (1.11–1.17)^∗∗^
Score in IGDS-SF9	REF	3.36 (2.97–3.79)^∗∗^	6.69 (5.86–7.64)^∗∗^
Score in depression^a^	REF	0.96 (0.92–1.01)	0.94 (0.91–0.98)
Score in anxiety^a^	REF	1.04 (1.03–1.06)^∗∗^	1.11 (1.09–1.13)^∗∗^
Score in stress^a^	REF	1.02 (1.00–1.04)^∗^	1.05 (1.03–1.07)^∗∗^
Score in PSQI	REF	2.62 (2.39–2.87)^∗∗^	5.56 (4.94–6.26)^∗∗^

*OR, odds ratio. ^*a*^Depression, anxiety, and stress were measured using Depression Anxiety Stress Scales. IGDS-SF9, Internet Gaming Disorder Scale-Short Form; PSQI, Pittsburgh Sleep Quality Index. ^∗^*p* < 0.05 and ^∗∗^*p* < 0.01.*

According to the suggested four different categories of gamers in previous studies, there were 2.4% addicted gamers, 3.9% problem gamers, 5.1% engaged gamers, and 88.6% normal gamers.

### Psychometric Findings From Rasch Analysis

#### Item Properties

**FIGURE 2:**
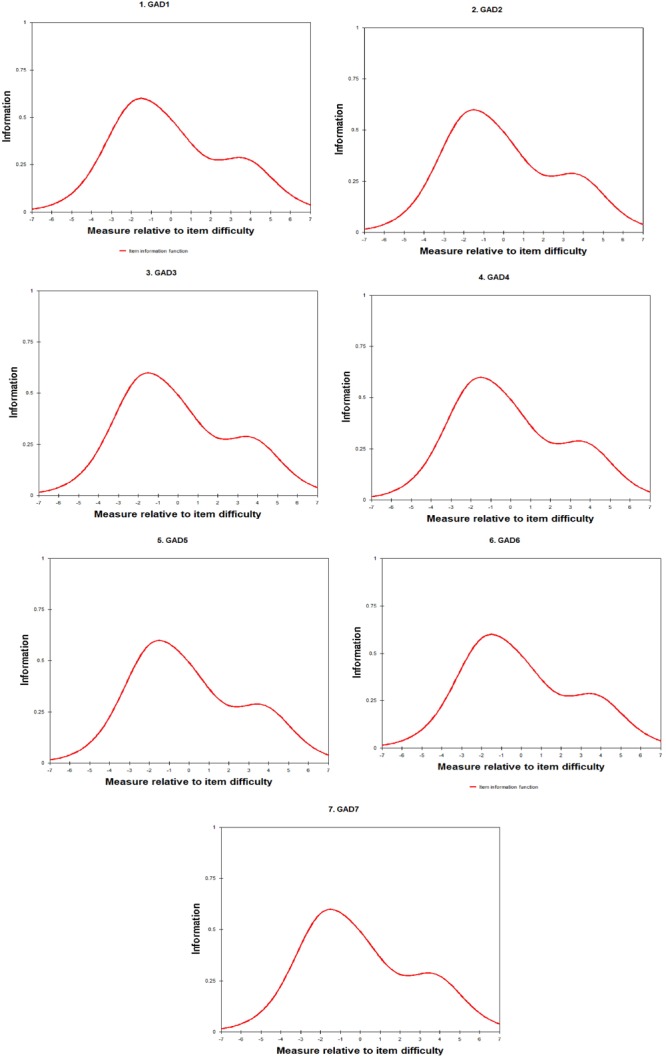
Item information function of Gaming Addiction Scale items 1 to 6.

Rasch fit statistics (Infit MnSq = 0.76 to 1.10; Outfit MnSq = 0.70 and 1.14), and DIF contrasts (range between -0.23 and 0.20 for gender; between -0.48 and 0.29 for time spent gaming online) were satisfactory. In addition, the Rasch analysis indicated that the range of difficulty for the items was between -0.82 and 0.59 (Table 2). Additionally, the local independence was supported by the low correlations between the item residuals (all *r* < 0.3). The IIF of GAS Items 1 and 6 is presented in Figure 2, and both items showed that the information retrieved from each item was within the range of -7 to 7 *logits* for respondents’ ability, where around -2 *logits* had the largest information.

#### Scale Properties

**FIGURE 3:**
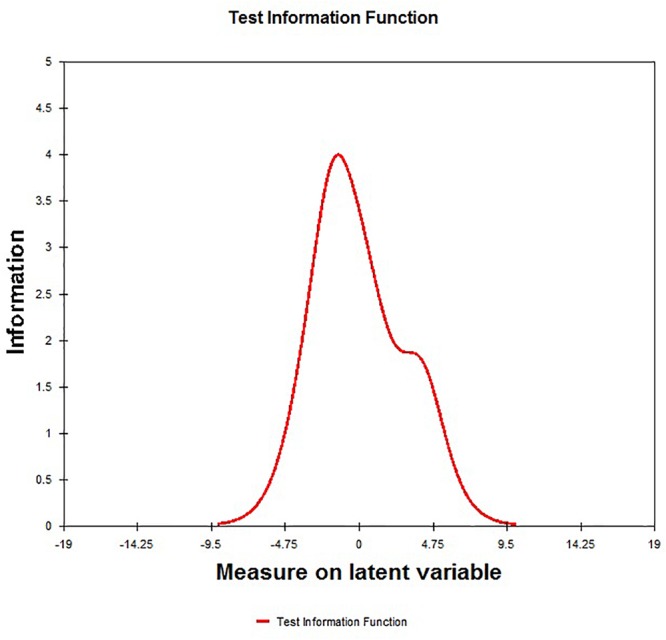
Test information function of Gaming Addiction Scale.

From the scale level, the results showed adequate separation reliability and index from Rasch (item separation reliability = 1.00, item separation index = 32.17, person separation reliability = 0.88, and person separation index = 2.53; Table 3). TIF is presented in Figure 3, and respondents’ ability around -2 *logits* had the largest information.

## Discussion

Using psychometric testing comprising classical test theory and modern test theory, the present study comprehensively and thoroughly demonstrated that the Persian GAS is an instrument suitable for assessing gaming addiction among Persian adolescents. Overall, the unidimensional structure of the GAS was supported by both CFA and the Rasch analysis results. The measurement invariance across gender and time spent on gaming was supported by the nested models in the MGCFA and the DIF in the Rasch analysis. As the psychometric properties of the Persian GAS were ascertained using both classical test theory and Rasch analysis, the psychometric results are robust to support the use of the Persian GAS in assessing gaming addiction among Iranian adolescents. More specifically, the evidence of the relationship between gaming addiction and poor academic achievement was found in one previous Iranian study (Haghbin et al., 2013) and these findings are now arguably stronger because the present study demonstrated that the Persian GAS is valid. Moreover, the LCA classified the study’s participants into three subgroups based on their risk for developing gaming addiction.

The psychometric results confirm previous studies on the 7-item GAS in that it had excellent internal consistency, good criterion-related validity with reliable external criteria (e.g., time on the gaming, depression, anxiety), and unidimensionality (Gaetan et al., 2014; Baysak et al., 2016; Khazaal et al., 2016). The measurement invariance of the GAS has been supported previously across two linguistic samples (French and Germany; Khazaal et al., 2016), and the present results additionally demonstrated that the GAS can be used to make invariant comparisons between genders and between different time on gaming. Therefore, healthcare providers need not to worry whether male and female respondents or heavy and non-heavy gamers interpret the GAS differently.

In terms of the criterion-related validity, the variables investigated showed different magnitudes in the associations. More specifically, strong associations were found between the GAS and the following variables: IGDS-SF9, PSQI, and weekly hours spent gaming online. Weak associations were found between the GAS and the following criteria: depression, anxiety, stress, and PedQL^TM^ 4.0 SF15. The magnitudes were anticipated because time spent on gaming, IGDS-SF9, and the GAS all *assess the concept of gaming* (Lemmens et al., 2009; Pontes and Griffiths, 2015; Wu et al., 2017). Sleep is directly related to gaming, and mental health or quality of life are indirectly related to gaming. Indeed, other studies have shown that the GAS has relatively weak association with mental health [*r* = 0.12 with worry (Gaetan et al., 2014); *r* = -0.14 with life satisfaction (Lemmens et al., 2009)] and time spent on playing games has a weak association with quality of life (*r* = 0.11; Dolatabadi et al., 2013).

In terms of the three subgroups with different risks of gaming addiction classified by LCA, they had significant differences in IGDS-SF9 scores, depression, anxiety, stress, and sleep quality indicating that the GAS has good criterion validity. The group at high risk of gaming addiction had severe IGD, high emotional distress (depression, anxiety, and stress), and low sleep quality. The findings correspond to results from previous studies in which impaired emotional distress (Weinstein et al., 2015; Pontes et al., 2016) and addiction to other behaviors (Rücker et al., 2015; Pontes, 2017) were associated with gaming addiction.

Given that the psychometric properties of the Persian GAS have now been established, researchers (in future studies) and healthcare providers can use the Persian GAS to assess and diagnose gaming addiction for Iranian adolescents. More specifically, the adolescents can be assessed or diagnosed using the seven criteria of salience, tolerance, mood, modification, withdrawal, relapse, conflict, and problems proposed by the DSM and other researchers (Griffiths, 2005; Griffiths and Davies, 2005; Lemmens et al., 2009). In other words, using the Persian GAS will help researchers and clinicians to better understand gaming addiction among adolescents using theory related to addiction. Also, the GAS was designed specifically for adolescents with its items corresponding to the developmental stage of an adolescent (Lemmens et al., 2009). For example, items were designed to relate to homework or parents. Consequently, the use of Persian GAS is fully focused on adolescents.

### Strength and Limitations

There were a number of strengths in the present study. First, it had a large sample size with males and females equally distributed. Therefore, the psychometric results are relatively stable. Second, the psychometric testing included both classical test theory and modern test theory. In other words, the psychometric results presented in this study were thorough, rigorous, and comprehensive. To the best of our knowledge, no previous studies have applied both theories to test the psychometric properties of the GAS. Third, and following on from the second strength, the study used several external criteria to examine the criterion-related validity of the GAS, which further strengthens the robustness of the psychometric testing. Finally, the translation procedure was standardized and appropriate, and ensured the linguistic validity of the Persian GAS.

There are some limitations in the present study. First, although the adolescent sample was large, it was not nationally representative. Therefore, the results using classical test theory might not be able to generalize to Iranian adolescents more generally. Second, all the external criteria used for criterion-related validity of the GAS were self-reported. Therefore, respondents might not correctly report in these external criteria because of social desirability or memory recall biases. Consequently, future studies using other objective measures are warranted. For example, sleep quality could be measured using an actigraph. Third, only the 7-item GAS was tested in the present study. Therefore, the psychometric properties of the 21-item GAS using modern test theory are at present uncertain. Therefore, future studies with Persian-speaking populations are recommended to use the 7-item version rather than the 21-item GAS. Additionally, the 21-item GAS needs to be further evaluated for its psychometric properties.

## Conclusion

In conclusion, the present study demonstrates that the Persian GAS is a reliable and valid instrument to assess the addiction to gaming among Persian-speaking adolescents. Given that the prevalence rates of online game addiction have become a serious public health issue worldwide, including Far East countries (Baysak et al., 2016), the need of a validated instrument assessing addiction to gaming are paramount. Therefore, the present study’s findings provide researchers and healthcare providers with a psychometrically robust instrument to use in their day-to-day practices and help raise the awareness of problematic gaming.

## Author Contributions

AP, C-YL, and VI initiated and conceived the study, analyzed the data, and drafted the manuscript. MG supervised the entire study. AP, AB, KÅ, and MG critically reviewed the manuscript and provided constructive comments.

## Conflict of Interest Statement

The authors declare that the research was conducted in the absence of any commercial or financial relationships that could be construed as a potential conflict of interest.
